# Wellbeing and distress in young people with chronic conditions: how do positive psychology variables relate to mental health outcomes?

**DOI:** 10.1080/21642850.2023.2274539

**Published:** 2023-11-06

**Authors:** Asha Parkinson, Barbara Mullan, Keely Bebbington, Elizabeth Davis, Claire Treadgold, Amy Finlay-Jones

**Affiliations:** a Telethon Kids Institute, Nedlands, Australia; b enAble Institute, School of Population Health, Curtin University, Bentley, Australia; c WACPRU, School of Population Health, Curtin University, Bentley, Australia; d Children’s Diabetes Centre, Telethon Kids Institute, The University of Western Australia, Perth, Australia.; e Centre for Child Health Research, University of Western Australia, Perth, Australia; f Department of Endocrinology and Diabetes, Perth Children’s Hospital, Perth, Australia.; g Division of Paediatrics, Medical School, The University of Western Australia, Perth, Australia.; h Starlight Children’s Foundation, Naremburn, NSW, USA; i Discipline of Paediatrics & Child Health, UNSW, Medicine & Health, University of New South Wales, Sydney, NSW, USA

**Keywords:** Positive psychology, chronic conditions, youth, wellbeing

## Abstract

**Objective:**

The aim of this study was to determine the unique and shared contributions of various positive psychology constructs (gratitude, optimism, hope, self-compassion, self-efficacy, and emotion regulation) to wellbeing and distress outcomes in young people living with a diverse range of chronic health conditions.

**Methods and Measures:**

169 Australians (84.0% female, mean age = 21.2) who reported living with a chronic physical condition completed a cross-sectional survey assessing wellbeing, distress, and each positive psychology variable. Two multiple regressions were used to determine the unique and shared contributions of the positive psychology variables to wellbeing and distress outcomes.

**Results:**

When considered alongside symptom severity, the variables explained 53.4% and 38.1% of variance in distress and wellbeing, respectively. Only optimism and self-efficacy accounted for unique and significant variance in the model predicting wellbeing, accounting for 6.1% and 4.6% of unique variance, respectively. For the distress model, optimism, self-compassion, and emotion regulation each accounted for significant variance. When considered alongside other variables, hope and gratitude did not contribute to either model.

**Conclusion:**

Findings suggest that individual positive psychology variables differentially contribute to wellbeing and distress outcomes in young people with chronic conditions. Optimism appears to account for unique variance in both outcomes, suggesting it may be a parsimonious target to promote complete mental health in this population.

## Introduction

The developmental stage between 16 and 25 years is a unique period characterised by a heightened risk for mental health challenges. For between 10 and 35% of youth (Adams et al., 2019; Eurostat, 2020), this risk is further magnified by the presence of a chronic condition. A chronic condition is any medical condition that persists for longer than 3 months, requires ongoing management, and impacts an individual’s functioning (Mokkink et al., 2008). Numerous chronic conditions may impact young adults including conditions as varied as type 1 diabetes, juvenile idiopathic arthritis, and cancer. While symptoms differ across conditions, common experiences include coping with fluctuating symptoms, adhering to treatment regimens, and navigating the impact of the condition on day-to-day activities (Denny et al., 2014; Jones et al., 2021).

Living with a chronic condition may attenuate a young person's ability to engage in the necessary activities to develop social and psychological resources that are protective for mental health, such as engaging in hobbies and socialising with peers (Denny et al., 2014; Emerson et al., 2012). In line with this, youth diagnosed with a chronic condition have a 51% percent increased risk of developing a mental health condition compared to their peers (Adams et al., 2019). Such co-morbid mental health conditions have consequences for treatment adherence and may lead to poorer disease prognosis over the lifespan (Cossu et al., 2017). Given this unique landscape of risk faced by youth with chronic conditions, there is a clear need to identify intervention targets that can promote wellbeing and bolster this population against mental health challenges.

### Positive psychology

Research suggests that positive psychology may be a useful approach for improving mental health outcomes for individuals with chronic conditions (Nierenberg et al., 2016). Instead of focusing on reducing psychopathology, a positive psychology approach involves fostering an individual’s psychological ‘assets’, such as optimism and gratitude, that both enhance wellbeing and act as a buffer against future experiences of distress (Seligman, 2002). As such, this approach may be suitable for promoting mental health despite the ongoing challenges associated with living with a chronic condition, such as unpredictable symptoms or acute health complications (Macaskill, 2016).

Meta-analyses of positive psychology interventions have demonstrated their ability to simultaneously reduce distress and promote wellbeing in various populations (Carr et al., 2020). However, a review by Ghosh and Deb (2017) of positive psychology interventions for populations with chronic illness reported less conclusive findings. The authors attributed this to the tendency for interventions to combine various techniques targeting a multitude of positive psychology constructs. Similarly, Ciarrochi et al. (2022) argue that research determining the effective components of positive psychology interventions has been stymied by poor delineation of which specific constructs are being targeted within interventions.

While interventions broadly promoting positive psychology constructs may improve mental health, such programs preclude exploration of which specific constructs contribute to these changes, in what contexts, and for whom. Of note, there have been calls for increased research focusing on single components within positive psychology interventions (Ghosh & Deb, 2017; Pawelski, 2020). Such research will allow the development of streamlined interventions targeting only the most effective constructs, given a particular population and intervention context (Kern et al., 2020).

In the current study, six positive psychology variables were explored based on evidence of their utility for improving mental health outcomes in youth with chronic conditions: gratitude, optimism, self-compassion, self-efficacy, emotion regulation, and hope. While each variable is considered to be an independent construct, there are clear similarities regarding how different variables are theorised to improve mental health. Although a comprehensive review is beyond the scope of this paper, each construct is briefly described below.

Optimism, self-efficacy, and hope each relate to an individual’s future expectancies. Optimism is defined as the tendency to expect positive future outcomes, while hope refers to an individual’s perception of their ability to achieve their future goals by developing and engaging with strategies towards achievement (Snyder et al., 1991). Self-efficacy encapsulates an individual’s perception of their ability to successfully perform actions that bring about desired outcomes (Bandura, 1994). Gratitude relates to an individual’s tendency to recognise positive outcomes, and to experience feelings of appreciation in response to this (McCullough et al., 2002). Each of these constructs has been consistently linked to adaptive mental health outcomes including wellbeing and reduced stress and depression (See Alarcon et al., 2013; Jackson et al., 2014; Wood et al., 2010).

Self-compassion and emotion regulation differ from the other constructs under exploration as they are specific to the context of difficult emotions or experiences. This focus is particularly important, given mounting criticism that positive psychology approaches do not acknowledge the fundamental role of negative emotions within the human experience (Kern et al., 2020). Emotion regulation involves the ability to recognise, understand, and accept the emotions one is experiencing, and to employ appropriate regulation strategies to moderate emotions based on personal or situational demands (Gratz & Roemer, 2004). Self-compassion is an adaptive way of relating to oneself when experiencing hardship by acknowledging unpleasant emotional responses and providing oneself with care and support (Neff, 2003a). Previous meta-analyses of self-compassion (Zessin et al., 2015) and emotion regulation (Kraiss et al., 2020) have demonstrated moderate to large associations with wellbeing and negative affect.

### Present study

To better understand how positive psychology constructs can benefit youth with chronic conditions, we must understand the shared and unique variance of each construct to mental health outcomes. Accordingly, the aim of this study is to investigate the unique contributions of six positive psychology constructs to two mental health outcomes, wellbeing and distress, in youth with chronic conditions. The decision to utilise measures of both positive and negative mental health was informed by positive psychology theory and the dual factor model of mental health, which suggests that positive mental health and mental illness are distinct but interrelated domains (Iasiello & Van Agteren, 2020). Given the exploratory nature of the research, no hypotheses were formed regarding the performance of individual predictors.

## Method

This study used a cross-sectional online survey design to collect data on positive psychology constructs and mental health outcomes in a sample of 169 young people living with chronic conditions.

### Data collection

Ethical approval for the study was obtained from the Human Ethics Office of the University of Western Australia (approval number: RA/4/20/5896). 169 Participants were recruited between 2020 and 2021. To be eligible to participate, an individual had to be aged between 16 and 25 years, living in Australia and self-report diagnosis with a chronic physical condition. Participants were excluded if their geolocation (as assessed by Qualtrics) was located outside of Australia, or if they reported being outside the age range or not having a diagnosis of a chronic condition. In addition, data from Qualtrics and analysis of responses to the free-text question ‘Please tell us, in your own words, what you find most difficult about having a chronic condition?’ was used to assess for fraudulent responses, which were then removed from the dataset. The survey took approximately 20 min to complete, and participants were offered a $10 voucher upon survey completion.

Recruitment primarily occurred via the social media of various organisations that support individuals with chronic conditions in Australia. Of the 18 organisations who participated in recruitment, two organisations were for general youth health, three represented chronic fatigue syndrome, two represented sleep disorders, and two represented genetic conditions. The remaining nine organisations each represented one of the following: asthma, alopecia, chronic pain, coeliac disease, cystic fibrosis, Chiari malformation, heart conditions, and epilepsy.

### Measures

#### Demographic questions

Participants reported age, gender, their Australian post-code, and diagnoses of both mental health and chronic physical condition via a combination of multiple choice and free-text responses.

#### Wellbeing

Wellbeing was assessed using the World Health Organisation Wellbeing Index (WHO-5), a validated measure of general wellbeing (World Health Organisation, 1998). For each of the 5 items, participants report how often in the last two weeks they have experienced a particular feeling (e.g. ‘I have felt cheerful and in good spirits’) from 0 (‘at no time’) to 5 (‘all the time’). A total score is calculated by summing the item scores and multiplying this sum by 4; final scores range from 0 to 100, representing the worst to the best possible wellbeing. Scoring classifications for the WHO-5 divide scores into good (≥50), low (between 29 and 49), and very low (≤28) wellbeing (Topp et al., 2015).

#### Distress

Distress was measured using the Kessler 10 Psychological Distress Scale (K-10), a widely validated, 10-item measure of broad psychological distress over the past 30 days (Kessler et al., 2002). Participants report how often they felt various negative emotions on a 5-point scale from 1 (‘none of the time’) to 5 (‘all of the time’). Item scores are summed to calculate a total score ranging from 10 to 50, with higher scores indicating higher levels of psychological distress. Scores on the K10 can be classified as low (10–15), moderate (16–21), high (22–29), and very high (30–50) levels of distress (Andrews & Slade, 2001).

#### Symptom severity

Physical symptom severity was assessed with a single-item numeric rating scale, ‘On a scale of 1–10, how severe would you say your current physical symptoms are?’ Possible responses ranged from ‘not severe at all’ (1) to ‘extremely severe’ (10). Single-item rating scales are commonly used to assess variables such as self-reported symptom severity, due to their applicability to multiple settings and low burden on the respondent (Hjermstad et al., 2011). Given the diversity of chronic conditions under investigation, a broad, single-item measure of symptom severity was considered appropriate for general applicability to all respondents.

#### Self-compassion

The Self-Compassion Scale – Short Form (SCS-SF) is a 12-item, self-report measure of an individual's level of self-compassionate behaviour (Raes et al., 2011). The SCS-SF conceptualises self-compassion as both the presence of positive self-responding and the absence of negative self-responding, with 6 items addressing each (Neff, 2003b). Participants report how often they commonly practise each self-response on a scale from 1 (‘almost never’) to 5 (‘almost always’). The mean item score is calculated to determine a total score between 1 and 5, with higher scores indicating higher levels of self-compassionate behaviour.

#### Hope

Hope was measured using the 12-item Adult Hope Scale (AHS) designed by Snyder et al. (1991). Participants respond to a number of statements about themselves (e.g. ‘I can think of many ways to get out of a jam’) on an 8-point scale from ‘Definitely false’ (1) to ‘Definitely true’ (8). After removing 4 ‘filler’ items, the remaining 8 items are summed to determine a total hope score.

#### Emotion regulation

The Difficulties in Emotion Regulation Short Form (DERS-SF) is a self-report measure of an individual's emotion regulation difficulties within six domains, (1) awareness of emotions, (2) clarity about emotions, (3) emotion acceptance, (4) access to adaptive emotion regulation strategies, (5) ability to engage in goal-directed behaviour during negative emotions, and (6) ability to manage impulses during negative emotions (Kaufman et al., 2016). Participants report how often they experience each difficulty on a scale from 1 (‘Almost never’) to 5 (‘Almost always’). Total scores range between 18 and 90, with higher scores indicating higher levels of emotion regulation difficulties.

#### Optimism

Optimism was measured using the 10-item Revised Life Orientation Test (LOT-R) by Scheier et al. (1994). Participants respond to questions about their outlook (e.g. ‘In uncertain times, I usually expect the best’) on a 5-point scale from 0 (‘Strongly Disagree’) to 4 (‘Strongly Agree’). Four of the 10 items are filler items which are not scored. Total scores range between 0 and 24, with higher scores indicating higher levels of optimism.

#### Self-efficacy

The Self-Efficacy for Managing Chronic Disease scale is a 6-item measure which assesses an individual's confidence in their ability to manage the effects of their condition on their life (Lorig et al., 2001). Participants report on their confidence in managing the impact of pain, distress, or fatigue on their daily life and goals on a 10-point scale from 1 (‘Not at all confident’) to 10 (‘Totally confident’). The mean score of the six items is calculated as a total score.

#### Gratitude

The Gratitude Questionnaire-Six-Item Form is a self-report measure assessing an individual's tendency to experience gratitude in their day-to-day life (McCullough et al., 2002). Participants rate their agreement for each item on a 7-point scale from 1 (‘Strongly Disagree’) to 7 (‘Strongly Agree’). The mean item score is calculated, with higher scores indicating a higher likelihood of experiencing gratitude.

## Results

### Descriptive statistics and demographic information

The final sample consisted of 169 Australians (142 female, 21 male, 5 trans or gender diverse, and 1 who declined to disclose) with a mean age of 21.2 years. Missing data analysis returned 3 participants each missing responses to a single item on one of the positive psychology measures. For each participant, a score for this item was calculated using the mean score of other scale items.

Participants reported a diverse range of chronic condition diagnoses (See Supplementary Table 1 for a list of all conditions) with the most commonly reported conditions being chronic pain (*n* = 65, 38.5%), asthma (*n* = 49, 9.0%), allergies (*n* = 37, 21.9%), and chronic fatigue syndrome (*n* = 35, 20.7%). The majority of participants (54.4%) reported having more than one chronic condition. On a scale of 1–10, participants reported a mean current symptom severity of 5.91 out of 10.

Participants generally reported low levels of wellbeing and high levels of distress. Based on WHO-5 scoring classifications, only 29.6% of participants reported ‘good’ wellbeing, while 34.9% and 35.5% met the criteria for low and very low wellbeing, respectively. Likewise, for distress, 8.9% of participants were categorised as having low distress, while 13.6%, 39.6%, and 37.9% were in the moderate, high, and very high distress categories. 55.0% of participants reported being diagnosed with at least one co-morbid mental health condition (Supplementary Table 1), with generalised anxiety disorder (*n* = 69, 40.8%) and major depressive disorder (*n* = 59, 34.9%) being the most common diagnoses. Additional chronic condition characteristics are summarised in Table 1. For all predictor and outcome variables, descriptive statistics and correlations are presented in Table 2.

**Table 1.** Demographic characteristics of participants (*N* = 169).

Characteristic	*n*	%
Number of chronic physical conditions		
1	77	45.6
2	39	23.1
3	23	13.6
4	14	8.3
5	6	3.6
6	2	1.2
7	3	1.8
8	2	1.2
9	2	1.2
10+	1	0.6
Time since diagnosis^a^		
4–6 months	4	2.4
7–11 months	7	4.1
1–2 years	18	10.7
3–4 years	34	20.1
≥5 years	97	57.4
Life-long (since birth)	8	4.7

^a^ Time since diagnosis missing for one participant. For participants with multiple conditions, time since diagnosis reflects the chronic condition of the longest duration.

**Table 2.** Description Statistics and Correlations for Key Variables.

Variable	1	2	3	4	5	6	7	8	9	10
1. Symptom Severity	–	.22*	−.33*	.35*	−.06	−.22*	.22*	−.16	−.42*	−.24*
2. Quantity of CPCs		–	−.20	.24*	−.01	.05	.07	−.20*	−.19	.02
3. Wellbeing			–	−.68*	.36*	.36*	−.31*	.48*	.49*	.24*
4. Distress				–	−.58*	−.43*	.62*	−.56*	−.48*	−.38*
5. Self-compassion					–	.41*	−.69*	.55*	.36*	.37*
6. Hope						–	−.47*	.43*	.48*	.59*
7. Emotion regulation							–	−.54*	−.46*	−.40*
8. Optimism								–	.43*	.46*
9. Self-efficacy									–	.38*
10. Gratitude										–
M	5.91	2.29	39.34	27.37	32.29	41.76	48.12	10.59	5.08	5.22
SD	2.14	1.79	20.05	7.98	9.51	9.98	13.17	5.22	2.09	1.07
α	–	–	.86	.89	.90	.86	.90	.83	.89	.81

Note: Numbers 1–9 in the horizontal header represent the variable in the vertical header with the same number. * *p* ≤ .01 (two-tailed).

### Multiple regression

Two hierarchical multiple regressions were conducted to investigate the contributions of the positive psychology constructs to variance in two mental health outcomes; wellbeing and distress. In each model, symptom severity was added as a covariate at step one of the regression, with positive psychology constructs (hope, self-efficacy, self-compassion, optimism, gratitude, and emotion regulation) added as predictors at the second step. All assumptions of the analysis technique were met.

The first model concerned the ability of the positive psychology variables to predict variance in wellbeing (Table 3). At step one, symptom severity accounted for a significant 10.8% of variance in wellbeing, *R*^2^ = 0.11, F (1, 167) = 20.16, *p* < .001. The addition of positive psychology variables at step two accounted for an additional 28.1% of variance in wellbeing, Δ*R*^2^ = 0.27, ΔF (6, 161) = 11.83, *p* < .001. In combination, the predictors and covariate significantly accounted for 38.1% of variance in wellbeing outcomes, *R*^2 ^= 0.38, F (7, 161) = 14.15, *p* < .001. In the final model, only symptom severity, optimism, and self-efficacy were significant predictors of wellbeing. The contributions of gratitude, self-compassion, hope, and emotion regulation were non-significant.

**Table 3.** Hierarchical regression results for wellbeing.

Variable	B	99% CI for B		SE B	*β*	sr^2^
		Lower	Upper			
Step 1						
Constant	57.51**	46.30	68.72	4.30		
Symptom Severity	−3.07**	−4.86	−1.29	0.69	−.33	.11
Step 2						
Constant	2.25	−35.91	40.40	14.64		
Symptom Severity	−1.82*	−3.52	−0.12	0.65	−.19	0.030
Self-Compassion	0.39	−0.12	0.80	0.19	0.16	0.012
Hope	0.25	−0.19	0.68	0.17	0.13	0.009
Emotion Regulation	0.25	−0.13	0.62	0.14	0.14	0.009
Optimism	1.25**	0.43	2.07	0.32	0.32	0.061
Self-Efficacy	2.67**	0.66	4.69	0.77	0.27*	0.043
Gratitude	−2.59	−6.52	1.33	1.51	−0.13	0.010

Note: sr^2^ = squared semi-partial correlation from SPSS. *p < .01; **p ≤ .001.

In the second model, distress was entered as the outcome variable (Table 4). At step one, the covariate of symptom severity accounted for a significant 12.1% of variance in distress, *R*^2^ = 0.12, F (1, 167) = 22.92, *p* < .001. At step two, the addition of the positive psychology constructs accounted for an additional 41.3% of variance, Δ*R*^2^ = 0.41, ΔF (6, 161) = 23.81, *p* < .001. The final model was able to significantly account for 53.4% of variance in distress outcomes, *R*^2 ^= 0.53, F (7, 161) = 26.37, *p* < .001. In the final model, symptom severity, optimism, self-compassion, and emotion regulation were significant (*p* < .01) predictors of distress, while hope, self-efficacy, and gratitude were non-significant.

**Table 4.** Hierarchical regression results for distress.

Variable	B	99% CI for B		SE B	β	sr^2^
		Lower	Upper			
Step 1						
Constant	19.71**	15.27	24.12	1.70		
Symptom Severity	1.30**	0.59	2.00	0.27	0.35	0.121
Step 2						
Constant	29.18**	16.00	42.37	5.06		
Symptom Severity	0.78**	0.19	1.36	0.23	0.21	0.034
Self-Compassion	−0.20*	−0.38	−0.03	0.07	−0.24	0.027
Hope	−0.05	−0.20	0.11	0.06	−0.06	0.002
Emotion Regulation	0.14*	0.07	0.27	0.05	0.22	0.023
Optimism	−0.35**	−0.64	−0.07	0.11	−0.23	0.030
Self-Efficacy	−0.32	−1.01	0.38	0.27	−0.81	0.004
Gratitude	0.15	−1.21	1.51	0.52	0.02	0.000

Note: sr^2^ = squared semi-partial correlation. *p ≤ .01; **p ≤ .001.

## Discussion

Positive psychology constructs demonstrate potential as intervention targets for improving the mental health of young people with chronic conditions. Unfortunately, conceptual overlap between different positive psychology constructs contributes to the ongoing lack of clarity about which of these constructs are likely to deliver the greatest benefits for mental health. The aim of this study was to determine which positive psychology constructs demonstrated unique predictive value for two mental health outcomes, wellbeing and distress, when considered simultaneously. Consistent with the literature, the combination of positive psychology variables accounted for a substantial proportion of variance in both wellbeing and distress outcomes in our sample of youth with chronic conditions. Specifically, it was found that positive psychology variables, in combination with symptom severity, accounted for 38.1% of the variance in wellbeing and 53.4% of the variance in distress.

While our convenience sample may not be representative of the cohort of young people with chronic conditions, the distribution of distress and wellbeing scores in the current study reinforce the need to address the unique mental health needs of young people living with chronic conditions. Over 77% of participants had scores that indicated high or very high distress, compared to 15% of young people aged 18–24 in Australian population norms (Australian Bureau of Statistics, 2018). The mean wellbeing score of 39.3 in the current sample is considerably lower than that reported in a similar sample of young adults with various chronic conditions (M = 56.4) (Halloran et al., 2021). As the data used by Halloran and colleagues was collected prior to COVID-19, it is plausible that mental health outcomes in our sample were affected by the impact of the pandemic. Prior work has demonstrated the considerable mental impact of the pandemic on Australians during the period of our data collection, particularly for those aged 18–24 years (Rossell et al., 2021). The effect on young adults with chronic conditions is likely to be particularly severe, due to the impact of decreased access to health services and increased social isolation when aiming to minimise contact with COVID-19 (Serlachius et al., 2020).

### Positive psychology constructs and mental health

Emotion regulation and self-compassion each contributed to unique variance in distress but were not found to be significant predictors of wellbeing, while self-efficacy contributed unique variance to wellbeing but not distress. Of note, only optimism (alongside symptom severity) was a significant predictor of both outcomes. These findings align with those of one prior study. Karademas (2007) explored how self-efficacy, optimism, stress, and use of positive and negative coping strategies differentially contributed to future positive and negative wellbeing (e.g. distress and anxiety) in a sample of 201 healthy adults. Similar to the current findings, self-efficacy was a significant predictor for positive wellbeing, but not for negative wellbeing. Furthermore, optimism significantly contributed to both positive and negative wellbeing (Karademas, 2007). The current study extends these findings by demonstrating common and specific predictors of mental health outcomes in the context of youth living with chronic conditions.

In addition to optimism, only self-compassion, and emotion regulation were found to be significant predictors of distress. In line with this, prior research has demonstrated that the relationships between self-compassion and emotion regulation with negative mental health indicators are of a higher magnitude compared to the relationships with positive mental health indicators (Bluth et al., 2017; Hu et al., 2014). This finding is congruent with the theoretical underpinnings of the variables. For example, self-compassion is conceptualised by Neff (2003a) as a method of self-responding when confronted with difficult experiences, and emotion regulation primarily relates to an individual’s ability to understand and control negative emotions. This suggests that these constructs may be more relevant to the experience of distress than that of wellbeing. Prior research suggests that responding to health challenges with self-compassion primarily leads to a reduction in negative affect and promotes adaptive coping strategies, without directly increasing positive affect (Sherman et al., 2019; Terry et al., 2013). Many challenges experienced by young people with chronic conditions, such as feeling different from peers or struggling to adhere to self-management of conditions, may lead to feelings of shame and act as a trigger for self-criticism or maladaptive emotion regulation strategies (Trindade et al., 2020). Learning to respond to such challenges with higher levels of self-compassion and emotion regulation may benefit long-term mental and physical health (Santoro et al., 2014).

In addition to symptom severity, self-efficacy and optimism were found to be significant predictors of wellbeing. In contrast to constructs such as self-compassion and emotion regulation which relate to one’s response to emotional challenges, self-efficacy beliefs are theorised to shape experience even if the absence of challenges, providing further support for why self-efficacy may be more closely related to wellbeing than the other variables investigated. For young people with chronic conditions, a higher level of self-efficacy is likely to promote self-care autonomy, prompting them to undertake the wellness maintenance behaviours that are required to manage their condition (Eassey et al., 2020). In addition, by promoting perseverance and a problem-solving attitude, higher levels of self-efficacy may attenuate the impact of a chronic condition on goal achievement in social, academic and self-development domains (Casier et al., 2013; Cramm et al., 2013).

The finding that the majority of predictors account for individual variance in only one, or none of, the models is not surprising, given the considerable empirical and theoretical overlap between variables (Martz & Livneh, 2016). This overlap will impact how each positive psychology construct performs when input into a multiple regressions model simultaneously. In the current study, neither gratitude nor hope emerged as significant predictors for wellbeing or distress, despite the demonstrated associations of these variables with mental health outcomes in prior research (Alarcon et al., 2013; Wood et al., 2010). This suggests a large degree of overlap between these variables and the other variables of interest. In addition, although these results are not incompatible with positive psychology theory, they do suggest that we may need to develop a more nuanced understanding of the way that individual constructs under the ‘positive psychology’ umbrella operate. *Why* each construct differentially related to specific mental health outcomes is an interesting line of inquiry for future work.

The finding that optimism was the only common predictor of wellbeing and distress is notable, particularly when considered in relation to self-efficacy and hope. While self-efficacy emerged as a specific predictor of wellbeing, hope did not significantly predict either outcome. While all three constructs are future-focused, self-efficacy and hope are focused on what is controllable, or what an individual can do to improve their situation (Rand, 2018). In contrast, an optimistic person holds positive expectancies for the future, without assigning themselves the responsibility to create these outcomes. The utility of optimism over self-efficacy and hope makes sense in the context of living with chronic conditions where symptom severity and prognosis may be out of an individual’s control, such as cancer or chronic fatigue. In this scenario, optimism may promote mental health by allowing an individual to believe that their situation will improve in the future, without expecting themselves to exert control over a potentially uncontrollable situation (Vollmann et al., 2014). In contrast, self-efficacy may be more important in cases where condition management has considerable behavioural elements, such as type 1 diabetes (Rassart et al., 2016). Ultimately, it is likely that the relative importance of each of these constructs will depend on the unique characteristics of an individual’s condition (Bonsaksen et al., 2014; Jackson et al., 2014).

### Implications

Our findings suggest that despite conceptual overlap, positive psychological variables are differentially related to wellbeing and distress in young people with chronic conditions. Given the finding that most positive psychology constructs only contributed to one of the two mental health outcomes, decisions regarding which constructs to target in an intervention should be consider based on the intervention context and aim. For example, targeting self-compassion and emotion regulation may be more useful for interventions that aim to reduce distress following diagnosis or illness progression, while self-efficacy and optimism may be more effective targets for interventions that aim to broadly improve wellbeing for all young people with chronic conditions. In addition, the finding that optimism predicted both wellbeing and distress suggests that this may be a parsimonious intervention target, with the ability to bring mental health improvements to both clinical and community populations.

### Limitations and future directions

The current findings should be interpreted with consideration of several limitations. Firstly, the cross-sectional nature of the study precludes any definitive conclusions regarding the direction of the relationships with wellbeing and distress. It is very plausible that these relationships are bidirectional. For example, a core feature of depressive symptomology is a sense of hopelessness, which likely undermines optimism, hope, and self-efficacy. In contrast, the experience of distress in response to one’s chronic condition may also lead to the development of positive psychology skills through the process of post-traumatic growth (Livneh, 2022). Further, the current findings do not provide any insight into the predictive value of these constructs over time. As the majority of existing prospective studies have explored individual positive psychology constructs in isolation, future research utilising longitudinal designs to explore the contributions of all constructs to mental health over time is warranted.

Additional limitations include that participants represent a convenience sample of young people who provided self-report data and self-identified their condition to be chronic. This may lead to under-representation of individuals who do not consider their condition to fit under this terminology, as well as the potential for participants to over or underestimate survey responses. Finally, while information on type and duration of chronic conditions has been reported in this study, the format of this data limited our ability to include it within the correlational and regression analyses. Future research with more robust exploration of the interplay between chronic conditions characteristics with mental health and positive psychology constructs is warranted. Despite this, a large number of chronic conditions are represented in the sample, strengthening the argument that these findings may be consistent across chronic condition groups.

### Conclusions

To the authors’ knowledge, this is the first study to explore how various positive psychology variables act as shared and unique predictors for both positive and negative mental health outcomes in youth with chronic conditions. Of the six positive psychology variables, self-efficacy and optimism emerged as predictors for wellbeing, while optimism, self-compassion, and emotion regulation were predictors for distress. Hope and gratitude did not significantly contribute to either model. These findings have potential implications regarding intervention development for this unique population.

## Data Availability

There is no dataset available for this study, as consent forms and participant information forms did not specify any data sharing would occur. As such, participants were not able to consent to the dataset being shared.

## References

[CIT0001] Adams, J. S., Chien, A. T., & Wisk, L. E. (2019). Mental illness among youth with chronic physical conditions. *Pediatrics*, *144*(1), e20181819. 10.1542/peds.2018-1819 31201229 PMC12930273

[CIT0002] Alarcon, G. M., Bowling, N. A., & Khazon, S. (2013). Great expectations: A meta-analytic examination of optimism and hope. *Personality and Individual Differences*, *54*(7), 821–827. 10.1016/j.paid.2012.12.004

[CIT0003] Andrews, G., & Slade, T. (2001). Interpreting scores on the Kessler Psychological Distress Scale (K10). *Australian and New Zealand Journal of Public Health*, *25*(6), 494–497. 10.1111/j.1467-842X.2001.tb00310.x 11824981

[CIT0004] Australian Bureau of Statistics. (2018). *National health survey: First Results, 2017–18—Australia*. Commonwealth of Australia. https://www.abs.gov.au/statistics/health/mental-health/mental-health/2017-18.

[CIT0005] Bandura, A. (1994). Self-efficacy. In V. S. Ramachaudran (Ed.), *Encyclopedia of human behavior* (Vol. 4, pp. 71–81). Academic Press.

[CIT0006] Bluth, K., Campo, R. A., Futch, W. S., & Gaylord, S. A. (2017). Age and gender differences in the associations of self-compassion and emotional well-being in a large adolescent sample. *Journal of Youth and Adolescence*, *46*(4), 840–853. 10.1007/s10964-016-0567-2 27632177 PMC5346326

[CIT0007] Bonsaksen, T., Fagermoen, M. S., & Lerdal, A. (2014). Trajectories of self-efficacy in persons with chronic illness: An explorative longitudinal study. *Psychology & Health*, *29*(3), 350–364. 10.1080/08870446.2013.856432 24219510

[CIT0008] Carr, A., Cullen, K., Keeney, C., Canning, C., Mooney, O., Chinseallaigh, E., & O’Dowd, A. (2020). Effectiveness of positive psychology interventions: A systematic review and meta-analysis. *The Journal of Positive Psychology*, *16*(6), 749–769. 10.1080/17439760.2020.1818807

[CIT0009] Casier, A., Goubert, L., Gebhardt, W. A., Baets, F. D., Aken, S. V., Matthys, D., & Crombez, G. (2013). Acceptance, well-being and goals in adolescents with chronic illness: A daily process analysis. *Psychology & Health*, *28*(11), 1337–1351. 10.1080/08870446.2013.809083 23781975

[CIT0010] Ciarrochi, J., Hayes, S. C., Oades, L. G., & Hofmann, S. G. (2022). Toward a unified framework for positive psychology interventions: Evidence-based processes of change in coaching, prevention, and training. *Frontiers in Psychology*, *12*, 809362. 10.3389/fpsyg.2021.809362 35222161 PMC8866971

[CIT0011] Cossu, G., Carta, M. G., Contu, F., Mela, Q., Demelia, L., Elli, L., & Dell’Osso, B. (2017). Coeliac disease and psychiatric comorbidity: Epidemiology, pathophysiological mechanisms, quality-of-life, and gluten-free diet effects. *International Review of Psychiatry*, *29*(5), 489–503. 10.1080/09540261.2017.1314952 28681625

[CIT0012] Cramm, J. M., Strating, M. M. H., Roebroeck, M. E., & Nieboer, A. P. (2013). The importance of general self-efficacy for the quality of life of adolescents with chronic conditions. *Social Indicators Research*, *113*(1), 551–561. 10.1007/s11205-012-0110-0 23874059 PMC3696170

[CIT0013] Denny, S., De Silva, M., Fleming, T., Clark, T., Merry, S., Ameratunga, S., Milfont, T., Farrant, B., & Fortune, S. A. (2014). The prevalence of chronic health conditions impacting on daily functioning and the association with emotional well-being Among a national sample of high school students. *Journal of Adolescent Health*, *54*(4), 410–415. 10.1016/j.jadohealth.2013.09.010 24210897

[CIT0014] Eassey, D., Reddel, H. K., Ryan, K., & Smith, L. (2020). ‘It is like learning how to live all over again’ A systematic review of people’s experiences of living with a chronic illness from a self-determination theory perspective. *Health Psychology and Behavioral Medicine*, *8*(1), 270–291. 10.1080/21642850.2020.1794879 34040872 PMC8143269

[CIT0015] Emerson, E., Llewellyn, G., Honey, A., & Kariuki, M. (2012). Lower well-being of young Australian adults with self-reported disability reflects their poorer living conditions rather than health issues. *Australian and New Zealand Journal of Public Health*, *36*(2), 176–182. 10.1111/j.1753-6405.2011.00810.x 22487354

[CIT0016] Eurostat. (2020). *Health variables of EU-SILC* [Data set]. Eurostat. Retrieved October 5, 2022, from https://ec.europa.eu/eurostat/databrowser/view/HLTH_SILC_25/default/table?lang=en&category=hlth.hlth_state.hlth_srcm

[CIT0017] Ghosh, A., & Deb, A. (2017). Positive psychology interventions for chronic physical illnesses: A systematic review. *Psychological Studies*, *62*(3), 213–232. 10.1007/s12646-017-0421-y

[CIT0018] Gratz, K. L., & Roemer, L. (2004). Multidimensional assessment of emotion regulation and dysregulation: Development, factor structure, and initial validation of the Difficulties in Emotion Regulation Scale. *Journal of Psychopathology and Behavioral Assessment*, *26*(1), 41–54. 10.1023/B:JOBA.0000007455.08539.94

[CIT0019] Halloran, J., McDermott, B., Ewais, T., Begun, J., Karatela, S., D'Emden, H., Corias, C., & Denny, S. (2021). Psychosocial burden of inflammatory bowel disease in adolescents and young adults. *Internal Medicine Journal*, *51*(12), 2027–2033. 10.1111/imj.15034 32840949

[CIT0020] Hjermstad, M. J., Fayers, P. M., Haugen, D. F., Caraceni, A., Hanks, G. W., Loge, J. H., … Kaasa, S.. (2011). Studies Comparing Numerical Rating Scales, Verbal Rating Scales, and Visual Analogue Scales for Assessment of Pain Intensity in Adults: A Systematic Literature Review. *Journal of Pain and Symptom Management*, *41*(*6*), 1073–1093. 10.1016/j.jpainsymman.2010.08.016 21621130

[CIT0021] Hu, T., Zhang, D., Wang, J., Mistry, R., Ran, G., & Wang, X. (2014). Relation between emotion regulation and mental health: A meta-analysis review. *Psychological Reports*, *114*(2), 341–362. 10.2466/03.20.PR0.114k22w4 24897894

[CIT0022] Iasiello, M., & Van Agteren, J. (2020). Mental health and/or mental illness: A scoping review of the evidence and implications of the dual-continua model of mental health. *Evidence Base*, *2020*(1), 1–45. 10.21307/eb-2020-001

[CIT0023] Jackson, T., Wang, Y., Wang, Y., & Fan, H. (2014). Self-efficacy and chronic pain outcomes: A meta-analytic review. *The Journal of Pain*, *15*(8), 800–814. 10.1016/j.jpain.2014.05.002 24878675

[CIT0024] Jones, A., Caes, L., McMurtry, C. M., Eccleston, C., & Jordan, A. (2021). Sociodevelopmental challenges faced by young people with chronic pain: A scoping review. *Journal of Pediatric Psychology*, *46*(2), 219–230. 10.1093/jpepsy/jsaa101 33211876

[CIT0025] Karademas, E. C. (2007). Positive and negative aspects of well-being: Common and specific predictors. *Personality and Individual Differences*, *43*(2), 277–287. 10.1016/j.paid.2006.11.031

[CIT0026] Kaufman, E. A., Xia, M., Fosco, G., Yaptangco, M., Skidmore, C. R., & Crowell, S. E. (2016). The difficulties in emotion regulation scale short form (DERS-SF): Validation and replication in adolescent and adult samples. *Journal of Psychopathology and Behavioral Assessment*, *38*(3), 443–455. 10.1007/s10862-015-9529-3 PMC12788809 41522882

[CIT0027] Kern, M. L., Williams, P., Spong, C., Colla, R., Sharma, K., Downie, A., Taylor, J. A., Sharp, S., Siokou, C., & Oades, L. G. (2020). Systems informed positive psychology. *The Journal of Positive Psychology*, *15*(6), 705–715. 10.1080/17439760.2019.1639799

[CIT0028] Kessler, R. C., Andrews, G., Colpe, L. J., Hiripi, E., Mroczek, D. K., Normand, S. L., Walters, E. E., & Zaslavsky, A. M. (2002). Short screening scales to monitor population prevalences and trends in non-specific psychological distress. *Psychological Medicine*, *32*(6), 959–976. 10.1017/S0033291702006074 12214795

[CIT0029] Kraiss, J. T., ten Klooster, P. M., Moskowitz, J. T., & Bohlmeijer, E. T. (2020). The relationship between emotion regulation and well-being in patients with mental disorders: A meta-analysis. *Comprehensive Psychiatry*, *102*, 152189. 10.1016/j.comppsych.2020.152189 32629064

[CIT0030] Livneh, H. (2022). Psychosocial adaptation to chronic illness and disability: An updated and expanded conceptual framework. *Rehabilitation Counseling Bulletin*, *65*(3), 171–184. 10.1177/00343552211034819

[CIT0031] Lorig, K. R., Sobel, D. S., Ritter, P. L., Laurent, D., & Hobbs, M. (2001). Effect of a self-management program on patients with chronic disease. *Effective Clinical Practice*, *4*(6), 256–262. http://europepmc.org/abstract/MED/11769298 11769298

[CIT0032] Macaskill, A. (2016). Review of positive psychology applications in clinical medical populations. *Healthcare*, *4*(3), 66. 10.3390/healthcare4030066 27618122 PMC5041067

[CIT0033] Martz, E., & Livneh, H. (2016). Psychosocial adaptation to disability within the context of positive psychology: Findings from the literature. *Journal of Occupational Rehabilitation*, *26*(1), 4–12. 10.1007/s10926-015-9598-x 26283187

[CIT0034] McCullough, M. E., Emmons, R. A., & Tsang, J. A. (2002). The grateful disposition: A conceptual and empirical topography. *Journal of Personality and Social Psychology*, *82*(1), 112–127. 10.1037/0022-3514.82.1.112 11811629

[CIT0035] Mokkink, L. B., Van Der Lee, J. H., Grootenhuis, M. A., Offringa, M., & Heymans, H. S. A. (2008). Defining chronic diseases and health conditions in childhood (0–18 years of age): National consensus in the Netherlands. *European Journal of Pediatrics*, *167*(12), 1441–1447. 10.1007/s00431-008-0697-y 18340463

[CIT0036] Neff, K. (2003a). Self-compassion: An alternative conceptualization of a healthy attitude toward oneself. *Self and Identity*, *2*(2), 85–101. 10.1080/15298860309032

[CIT0037] NEFF, K. D. (2003b). The development and validation of a scale to measure self-compassion. *Self and Identity*, *2*(*3*), 223–250. 10.1080/15298860309027

[CIT0038] Nierenberg, B., Mayersohn, G., Serpa, S., Holovatyk, A., Smith, E., & Cooper, S. (2016). Application of well-being therapy to people with disability and chronic illness. *Rehabilitation Psychology*, *61*(1), 32–43*.* 10.1037/rep0000060 26881305

[CIT0039] Pawelski, J. O. (2020). The elements model: Toward a new generation of positive psychology interventions. *The Journal of Positive Psychology*, *15*(5), 675–679. 10.1080/17439760.2020.1789710

[CIT0040] Raes, F., Pommier, E., Neff, K. D., & Van Gucht, D. (2011). Construction and factorial validation of a short form of the Self-Compassion Scale. *Clinical Psychology & Psychotherapy*, *18*(3), 250–255. 10.1002/cpp.702 21584907

[CIT0041] Rand, K. L. (2018). Hope, self-efficacy, and optimism conceptual and empirical differences. In M. W. Gallagher & S. J. Lopez (Eds.), *The Oxford handbook of hope* (Vol. 1, pp. 45–58). Oxford University Press.

[CIT0042] Rassart, J., Luyckx, K., Oris, L., Goethals, E., Moons, P., & Weets, I. (2016). Coping with type 1 diabetes through emerging adulthood: Longitudinal associations with perceived control and haemoglobin A1c. *Psychology & Health*, *31*(5), 622–635. 10.1080/08870446.2016.1144753 26902672

[CIT0043] Rossell, S. L., Neill, E., Phillipou, A., Tan, E. J., Toh, W. L., Van Rheenen, T. E., … Meyer, D. (2021). An overview of current mental health in the general population of Australia during the COVID-19 pandemic: Results from the COLLATE project. *Psychiatry Research*, *296*, 113660. 10.1016/j.psychres.2020.113660 33373808 PMC7836867

[CIT0044] Santoro, M. S., Van Liew, C., Cronan, T. A., Franks, H. M., Adams, R. N., Roesch, S. C., Wooldridge, J. S., & Tomita, M. (2014). Physical function and quality of well-being in fibromyalgia: The applicability of the goodness-of-fit hypothesis. *Health Psychology and Behavioral Medicine*, *2*(1), 496–508. 10.1080/21642850.2014.905205 25750797 PMC4346089

[CIT0045] Scheier, M. F., Carver, C. S., & Bridges, M. W. (1994). Distinguishing optimism from neuroticism (and trait anxiety, self-mastery, and self-esteem): A re-evaluation of the Life Orientation Test. *Journal of Personality and Social Psychology*, *67*(6), 1063–1078. 10.1037/0022-3514.67.6.1063 7815302

[CIT0046] Seligman, M. E. (2002). Positive psychology, positive prevention, and positive therapy. In C. R. Snyder & S. J. Lopez (Eds.), *Handbook of positive psychology*, (pp. 3–12). Oxford University Press.

[CIT0047] Serlachius, A., Badawy, S. M., & Thabrew, H. (2020). Psychosocial challenges and opportunities for youth with chronic health conditions during the COVID-19 pandemic. *JMIR Pediatrics and Parenting*, *3*(2), e23057. 10.2196/23057 33001834 PMC7553787

[CIT0048] Sherman, K. A., Roper, T., & Kilby, C. J. (2019). Enhancing self-compassion in individuals with visible skin conditions: randomised pilot of the ‘My Changed Body’ self-compassion writing intervention. *Health Psychology and Behavioral Medicine*, *7*(1), 62–77. 10.1080/21642850.2019.1587298 34040839 PMC8114345

[CIT0049] Snyder, C. R., Harris, C., Anderson, J. R., Holleran, S. A., Irving, L. M., Sigmon, S. T., Yoshinobu, L., Gibb, J., Langelle, C., & Harney, P. (1991). The will and the ways: Development and validation of an individual-differences measure of hope. *Journal of Personality and Social Psychology*, *60*(4), 570–585. 10.1037/0022-3514.60.4.570 2037968

[CIT0050] Terry, M. L., Leary, M. R., Mehta, S., & Henderson, K. (2013). Self-Compassionate reactions to health threats. *Personality and Social Psychology Bulletin*, *39*(7), 911–926. 10.1177/0146167213488213 23813424

[CIT0051] Topp, C. W., Østergaard, S. D., Søndergaard, S., & Bech, P. (2015). The WHO-5 Well-Being Index: A systematic review of the literature. *Psychotherapy and Psychosomatics*, *84*(3), 167–176. 10.1159/000376585 25831962

[CIT0052] Trindade, I. A., Ferreira, C., & Pinto-Gouveia, J. (2020). Shame and emotion regulation in inflammatory bowel disease: Effects on psychosocial functioning. *Journal of Health Psychology*, *25*(4), 511–521. 10.1177/1359105317718925 28810497

[CIT0053] Vollmann, M., Scharloo, M., Langguth, B., Kalkouskaya, N., & Salewski, C. (2014). Illness representations as mediators of the relationship between dispositional optimism and depression in patients with chronic tinnitus: A cross-sectional study. *Psychology & Health*, *29*(1), 81–93. 10.1080/08870446.2013.828294 23937149

[CIT0054] Wood, A. M., Froh, J. J., & Geraghty, A. W. A. (2010). Gratitude and well-being: A review and theoretical integration. *Clinical Psychology Review*, *30*(7), 890–905. 10.1016/j.cpr.2010.03.005 20451313

[CIT0055] World Health Organization. (1998). *The use of well-being measures in primary health care - the DepCare project*. WHO Regional Office of Europe. https://www.euro.who.int/__data/assets/pdf_file/0016/130750/E60246.pdf

[CIT0056] Zessin, U., Dickhäuser, O., & Garbade, S. (2015). The relationship between self-compassion and well-being: A meta-analysis. *Applied Psychology: Health and Well-Being*, *7*(3), 340–364. 10.1111/aphw.12051 26311196

